# Policy Series: Aging and Health Policy Update: A View From Washington

**DOI:** 10.1093/geroni/igaf122.902

**Published:** 2025-12-31

**Authors:** Brian Lindberg, Patricia D’Antonio

## Abstract

Leading policy advocates will present their findings and viewpoints regarding aging and health care policy during a divided Congress. Issues will include behavioral health, social isolation, health care workforce, telehealth, hospice policy, elder justice and nursing home care, home and community-based services, and Medicare and Medicaid.

